# Precision pharmacotherapy of atomoxetine in children with ADHD: how to ensure the right dose for the right person?

**DOI:** 10.3389/fphar.2024.1484512

**Published:** 2024-10-29

**Authors:** Hong-Li Guo, Jian Huang, Jie Wang, Lin Fan, Yue Li, Dan-Dan Wu, Qian-Qi Liu, Feng Chen

**Affiliations:** ^1^ Pharmaceutical Sciences Research Center, Department of Pharmacy, Children’s Hospital of Nanjing Medical University, Nanjing, China; ^2^ Department of Children Healthcare, Children’s Hospital of Nanjing Medical University, Nanjing, China

**Keywords:** atomoxetine, attention deficit/hyperactivity disorder (ADHD), children, therapeutic drug monitoring (TDM), CYP2D6, inter-individual variability, precision pharmacotherapy, biomarkers

## Abstract

Non-stimulant atomoxetine is recognized in various current clinical guidelines as an important alternative to stimulants for the pharmacological treatment of attention deficit/hyperactivity disorder (ADHD) in children. While its efficacy and tolerability for core symptoms are established, there is considerable inter-individual variability in response and exposure, highlighting the need for personalized dosing. In this review, we evaluated existing studies and summarized comprehensive evidence supporting the clinical implementation of therapeutic drug monitoring (TDM) and personalized dosing of atomoxetine, organized around a series of logically structured questions. Although there are notable gaps in achieving personalized dosing across multiple critical elements, the available evidence is helpful to endorse personalized dose adjustments based on TDM and *CYP2D6* genotyping “whenever possible.” We advocate for ongoing improvement and enhancement in clinical practice. Future advancements will rely on a deeper understanding of ADHD, facilitating more precise diagnoses and personalized treatment strategies.

## 1 Introduction

Attention deficit-hyperactivity disorder (ADHD) is identified by symptoms of hyperactivity and impulsivity, inattention, or a combination of these, which exceed the expected development level and interfere with daily functioning (Posner et al., 2020; Fu et al., 2021). It is one of the most frequently diagnosed neurodevelopmental disorder in children, with up to 70% of cases showing symptoms that persist into adulthood. Approximately 5% of children and adolescents, as well as 2.5% of adults worldwide, are affected by ADHD, and the overall prevalence of the disorder has remained consistent over the past 3 decades (Posner et al., 2020; Faraone et al., 2015; Faraone et al., 2024). However, there has been a significant rise in new ADHD diagnoses and reported prevalence during the COVID-19 pandemic, particularly in countries like Finland (Auro et al., 2024) and the United States (Danielson et al., 2024; QuickStats, 2024), alongside a global increase in ADHD symptoms (Rogers and MacLean, 2023). This trend suggests that more individuals may now be eligible for treatment with atomoxetine in the aftermath of pandemic.

Treatment for individuals with ADHD may include pharmacological, non-pharmacological, or a combination of both approaches (Table 1). The available medications consist of stimulants, such as methylphenidate and amphetamines, as well as non-stimulants, including atomoxetine, extended-release clonidine, and guanfacine (Cortese, 2020). Currently, the process of selecting the most suitable medication for each patient is largely based on a trial-and-error, as our understanding of the neurobiology underlying ADHD is still inadequate to guide medication choices (Cortese, 2020).

**TABLE 1:** Treatment ladders and sequencing of medications (Coghill et al., 2023; Van Vyve et al., 2024).

Country (year)	Age	Treatment recommendation	Sequencing of medication
Spain (2017)	<6 years	Medication not recommended	N/A
	6–18 years	1st Psychological of pedagogical treatment/academic support 2nd Medication only recommended if 1st does not work, or in severe cases	No order specified medications recommended: methylphenidate, lisdexamfetamine, guanfacine and atomoxetine
United Kingdom (2018)	<5 years	ADHD-focused group parent training. Medication treatment not recommended	N/A
	6–12 years	1st: ADHD-specific information and support. 2nd: If persistent and significant impairment in at least one domain of life: offer medication. If comorbid oppositional defiant disorder or conduct disorder: add in a parent training program	1st methylphenidate 2nd lisdexamfetamine, (consider dexamphetamine if lisdexamfetamine not well tolerated) 3rd atomoxetine or guanfacine
	13–18 years	1st: Medication 2nd: If symptoms still impairing in at least one domain of life after medication treatment: offer cognitive behavioral therapy	
Canada (2018)	-	Psychosocial interventions for preschoolers	1st long-acting stimulants 2nd Atomoxetine, Guanfacine XR and short/intermediate acting psychostimulants 3rd bupropion, clonidine, imipramine and modafinil
German (2018)	<6 years	1st psychoeducation (patient/parents/educators) 2nd psychosocial interventions 3rd pharmacotherapy only by a physician with specialized knowledge in behavioral disorders in this age group Pharmacological treatment not recommended for <3 years	N/A
	6–18 years	Moderate to severe ADHD: Medication Mild to moderate ADHD: Psychological treatment	1st stimulants 2nd atomoxetine or guanfacine
Dutch (2019)	<6 years	1st Parent/teacher training; medication only considered in case of non-response to parent/teacher training	N/A
	6–12 years	1st psychoeducation (parents/teachers) 2nd Without behavioral problems: Mild: parent and/or teacher training; Moderate/severe: monotherapy: parent/teacher training OR pharmacotherapy With behavioral problems: Mild/moderate: parent and/or teacher training; Severe: combination therapy 3rd switch agent or combination therapy3rd switch agent or combination therapy	1st methylphenidate, preferably long-acting agents 2nd lisdexamfetamine or dexamfetamine 3rd atomoxetine or guanfacine (reserved for specialists) 4th other drugs such as clonidine or nortriptyline (reserved for specialists)
	13–18 years	1st psychoeducation (patient/parents/teachers) 2nd Mild: CBT with involvement of parents/teachers. Moderate/severe: Monotherapy CBT with or without parent/teacher training OR pharmacotherapy 3rd switching to another pharmacological agent or combination therapy	
Belgium (2021)	<6 years	1st psychoeducation (parents/teachers) 2nd parent/teacher training 3rd referral to specialist	N/A
	6–12 years	1st psychoeducation (parents/teachers) 2nd Without behavioral problems: Mild, parent training; Moderate/severe, monotherapy, pharmacological treatment. With behavioral problems: Mild/moderate, parent/teacher training; Severe, combination therapy 3rd combination therapy 3rd combination therapy	1st methylphenidate, preferably long-acting agents 2nd lisdexamfetamine or dexamfetamine 3rd atomoxetine or guanfacine 4th other drugs such as clonidine or nortriptyline (reserved for specialists)
	12–18 years	1st psychoeducation (patient/parents/teachers) 2nd Mild, CBT with involvement of parents/teachers Moderate/severe: monotherapy, pharmacotherapy OR CBT 3rd switching to CBT, pharmacological treatment, or combination therapy	
Denmark (2021)	6–18 years	1st psychological and/or educational interventions 2nd pharmacological treatment	1st methylphenidate (either short or long acting) or lisdexamfetamine/dexamfetamine or atomoxetine 2nd guanfacine or atomoxetine
China (2020) (Subspecialty Group of D, 2020)	4–6 years	Psychoeducation, cognitive behavioral therapy, special education and functional training	N/A
	>6 years	Combined treatment with drug therapy and non-drug therapy	First line: methylphenidate and atomoxetine Others: clonidine, guanfacine

In clinical settings, the American Academy of Pediatrics recommends stimulants, atomoxetine, extended-release guanfacine, and extended-release clonidine for children aged 6–11 years, listed in order of the strength of evidence. Similarly, the National Institute for Health and Care Excellence (NICE) advises starting medication for children aged 5 and older and young people with methylphenidate, lisdexamfetamine (or dexamphetamine if lisdexamfetamine causes unacceptable side effects), atomoxetine or guanfacine, ranked by preference. According to the ADHD German Guidelines, second-line pharmacotherapy for children aged 6 and older and young people with mild-to-moderate ADHD should include stimulants, atomoxetine or guanfacine, also in descending order of preference, following psychoeducation. For cases of moderate-to-severe ADHD, however, stimulants, atomoxetine or guanfacine should be prioritized as the first-line medication after psychoeducation (Cortese, 2020; Coghill et al., 2023). Despite these guidelines, a lack of high-quality, long-term evidence is evident in clinical guidance (Kazda et al., 2024). A one-dose-fits-all medication approach may not be appropriate for individuals with ADHD, and clinicians often face the significant challenge of tailoring pharmacological formulations and doses to align with each patient’s biological characteristics and social needs.

**FIGURE 1:**
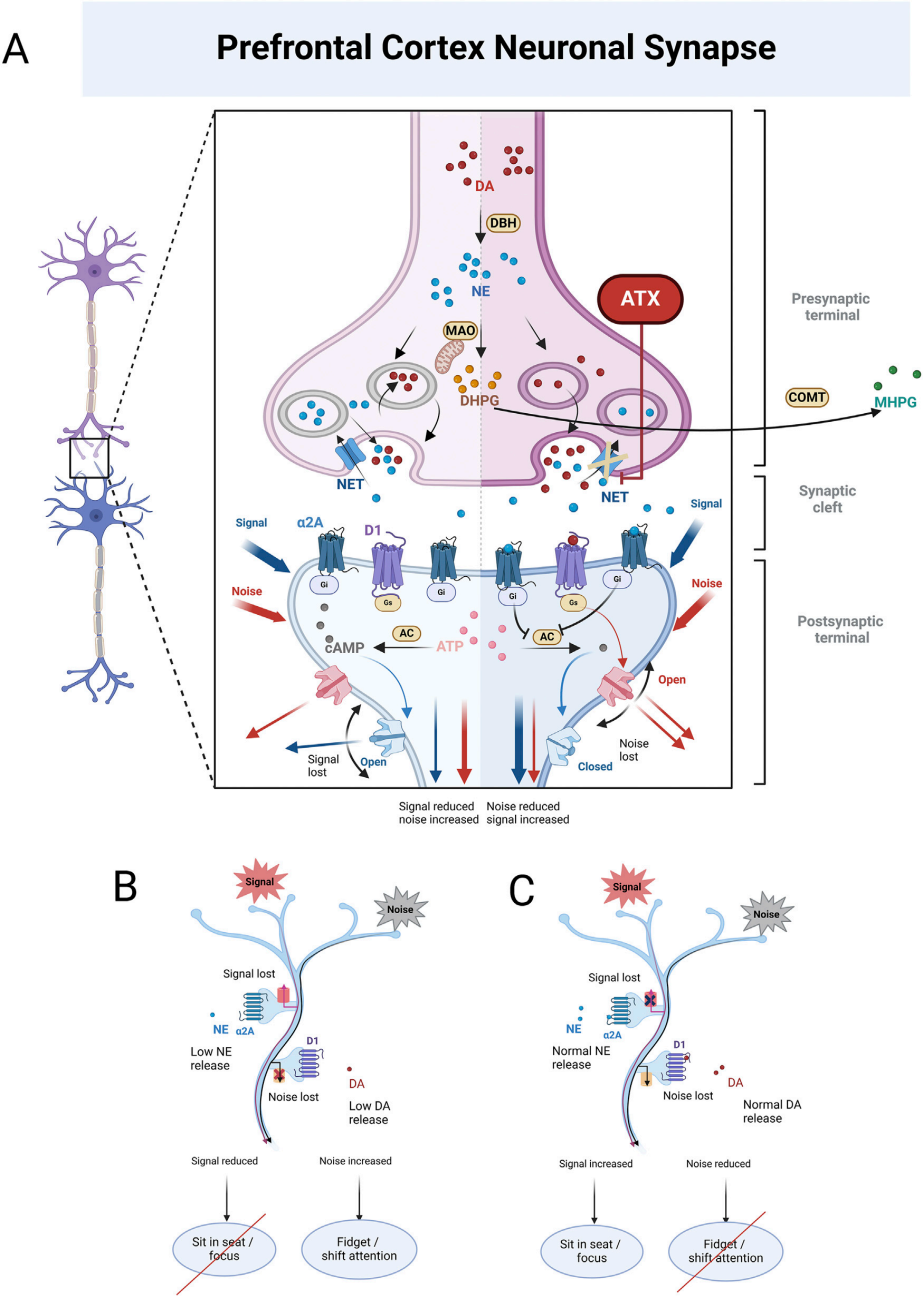
**(A)** illustrates the enzymatic conversion of dopamine (DA) into norepinephrine (NE) mediated by dopamine β-Hydroxylase (DBH). Once formed, NE is metabolized on the mitochondrial membrane by monoamine oxidase (MAO), generating 3,4-dihydroxy phenylethylene glycol (DHPG). DHPG is then further metabolized extracellularly by catechol-O-methyltransferase (COMT), resulting in the production of 3-methoxy-4-hydroxyphenylglycol (MHPG). At low to moderate concentrations, NE binds to α2A receptors, which activates G proteins associated with these receptors. These activated G proteins inhibit adenylyl cyclase (AC), the enzyme responsible for converting adenosine triphosphate (ATP) into cyclic adenosine monophosphate (cAMP). This reduction in cAMP levels leads to the closure of hyperpolarization-activated cyclic nucleotide-gated (HCN) channels, which are responsible for signal leakage, thereby enhancing the surviving signal. In contrast, at low to moderate DA concentrations, DA binds to D1 receptors, reducing interference noise in the brain. The left panel of **(A)** illustrates the state of neurons in the prefrontal cortex (PFC) of individuals with ADHD. In this context, both NE and DA levels in the synaptic cleft are low, impairing their ability to perform their respective functions. This deficiency leads to reduced signals and increased noise within the brain. Conversely, the right panel of **(A)** depicts individuals with ADHD after receiving atomoxetine (ATX) treatment. ATX selectively inhibits NET, preventing the reuptake of NE from the synaptic cleft back into the presynaptic terminal. This inhibition results in higher synaptic concentrations of NE and, subsequently, lower intra-neuronal NE levels. The decrease in intra-neuronal DHPG levels contributes to an increase in NE concentrations in the synaptic cleft. Importantly, the PFC has low levels of the dopamine transporter (DAT), responsible for DA reuptake. As a result, DA in this brain region is predominantly inactivated by NET inhibition, leading to elevated levels of both DA and NE in the PFC. This enables NE to effectively bind to α2A receptors, and DA to D1 receptors, allowing them to fulfill their functions as previously described. **(B)** illustrates the PFC condition in individuals with ADHD, showing that lower levels of NE and DA result in the ineffective receptor binding, which ultimately impairs their functions. **(C)** illustrates the PFC condition in individuals with ADHD when NE and DA levels are normal. In this scenario, NE binds to α2A receptors, and DA binds to D1 receptors, enabling NE to effectively enhance signaling while DA successfully reduces noise.

Approved by the US Food and Drug Administration (FDA) in 2002 as the first non-stimulant medication for the treatment of ADHD in children over 6 years and adults, atomoxetine functions as a selective inhibitor of the presynaptic noradrenaline transporter, thereby extending the activity of noradrenaline in the synaptic cleft (Figure 1) (Garland and Kirkpatrick, 2004). According to European and North American ADHD guidelines, atomoxetine is typically used as a second or third-line treatment (Coghill et al., 2023). However, in countries like China and Japan, it is regarded as a first-line option, equivalent to stimulants (Fu et al., 2023). It is also important to recognize that some patients discontinue treatment prematurely due to inadequate titration, lack of clinical monitoring, or insufficient ongoing evaluations (Vertessen et al., 2024). Consequently, regardless of its classification as a first, second, or third-line medication, if we consider the choice of atomoxetine for ADHD as an integrative, evidence-based decision for specific patients, the critical question becomes how to personalize the dosage regimen, given the individual differences in treatment response.

Despite the existence of evidence-based guidelines, a notable gap remains between these guidelines and their practical application in clinical settings, leading to uncertainty regarding the optimal utilization of the ADHD medication (Table 2). While less common, there are guidelines that offer specific recommendations for tailoring dosage regimen of atomoxetine (Hiemke et al., 2018; Brown et al., 2019). These guidelines suggest reference ranges for blood drug concentrations aligned with the timing of blood sampling and dosing schedules, which can help enhance clinical efficacy and manage adverse reactions. However, the low level of supporting evidence (Hiemke et al., 2018) and broad reference ranges present further challenges to implementing these guidelines. Thankfully, these recommendations have attracted considerable attention and have prompted significant advancements in this field.

**TABLE 2:** Recommended therapeutic reference ranges, elimination half-life (*t*_1/2_) ranges, levels of recommendation to use TDM from AGNP and pharmacogenomics guidelines from PharmGKB.

Drugs and active metabolites	Therapeutic reference range	*t*1/2 (h)	Level of TDM	Pharmacogenomics (from PharmGKB)
Methylphenidate	6–26 ng/mL 2 h after 20 mg IR or 4–6 h after 40 mg XR formulations	2	3	The DPWG Guideline methylphenidate state that no interaction was found between the *CYP2D6* and *COMT* genes and methylphenidate. (https://www.pharmgkb.org/chemical/PA450464/guidelineAnnotation/PA166182808-PA166264901)
Dexmethylphenidate	13–23 ng/mL 4 h after 20 mg	2	3	N/A
lisdexamfetamine	N/A	11.2^a^	N/A	N/A
Atomoxetine	200–1 000 ng/mL 60–90 min after intake of 1.2 mg/kg/day	2–5	3	The CPIC Dosing Guideline for atomoxetine provides therapeutic recommendations for CYP2D6 ultrarapid, normal, intermediate, and poor metabolizer, which includes guidance for plasma drug concentration testing, as a means to estimate atomoxetine exposure, if no clinical response and in the absence of adverse events after 2 weeks of therapy. (https://www.pharmgkb.org/chemical/PA134688071/guidelineAnnotation/PA166181885) The DPWG Guideline for atomoxetine states for CYP2D6 ultrarapid metabolizers, to be alert to reduced efficacy of atomoxetine or select an alternative drug as a precaution. Be alert to side effects in CYP2D6 poor metabolizers (https://www.pharmgkb.org/chemical/PA134688071/guidelineAnnotation/PA166104989)
Guanfacine	N/A	17.3^a^	N/A	N/A
Clonidine	N/A	13^b^	N/A	There are currently no dosing recommendations for clonidine based on CYP2D6 genotype and DPWG suggest clonidine as possible alternative for atomoxetine in variant CYP2D6 metabolizers. (https://www.pharmgkb.org/chemical/PA449051/guidelineAnnotation/PA166182818)
Nortriptyline^c^	70–170 ng/mL	18–44	1	The CPIC Dosing Guideline update for nortriptyline recommends a 25% dose reduction for CYP2D6 intermediate metabolizers. For CYP2D6 ultrarapid or poor metabolizers, an alternative drug should be considered. If nortriptyline is warranted, consider a 50% dose reduction in CYP2D6 poor metabolizers. (https://www.pharmgkb.org/chemical/PA450657/guidelineAnnotation/PA166104998) The DPWG Guideline for nortriptyline recommends a dose reduction for CYP2D6 poor or intermediate metabolizer patients. For CYP2D6 ultrarapid metabolizers, select an alternative drug or use 1.7 times the standard dose. Monitoring of nortriptyline and 10-hydroxynortriptyline plasma concentrations is recommended. (https://www.pharmgkb.org/chemical/PA450657/guidelineAnnotation/PA166104961)

Notes:

^a^ *t*_1/2_ data of lisdexamfetamine and guanfacine (extended-release tablet) are from the report by Roesch et al. (2013).

^b^ *t*_1/2_ data of clonidine is from the study by Amna et al. (2024).

^c^ The TDM, and pharmacogenomics guidelines are used for anti-depression. N/A, not available. IR, immediate release; XR, retarded formulations.

In this review, we focus on the personalized dosing of atomoxetine. We will apply the framework established by Beumer et al. (2019) to systematically evaluate the available published studies, compiling extensive evidence for the clinical implementation of therapeutic drug monitoring (TDM) and personalized dosing of atomoxetine through a series of logically structured questions.

## 2 Body weight (BW)-based dosing strategy: one dose fits all?

Currently, the dosing of atomoxetine is primarily based on the BWs of children (Farhat et al., 2022). For those weighing up to 70 kg, the recommended initial total daily dose is approximately 0.5 mg/kg, which can be increased after a minimum of 3 days to reach a target dose of approximately 1.2 mg/kg. This can be administered as a single daily dose in the morning (*q.m.*) or divided into doses taken in the morning and late afternoon/early evening doses (*b.i.d.*) (Farhat et al., 2022). It's important to highlight that doses exceeding 1.2 mg/kg/day have not shown additional benefit (Brown et al., 2016). Indeed, for children and adolescents, the maximum total daily dose should not exceed 1.4 mg/kg or 100 mg, whichever is less.

Additionally, for those taking strong CYP2D6 inhibitors (such as paroxetine, fluoxetine, and quinidine) (Ring et al., 2002), or identified as CYP2D6 poor metabolizers (PMs), atomoxetine should also be started at 0.5 mg/kg/day. The dose may be increased to the typical target of 1.2 mg/kg/day only if there is no improvement after 4 weeks and the initial dose is well tolerated.

For children and adolescents weighing over 70 kg, the recommended initial total daily dose of atomoxetine is 40 mg, consistent with adult dosing guidelines. This dosage may be increased after a minimum of 3 days to reach a target total daily dose of about 80 mg. The medication can be administered either as a single daily dose in the morning or divided into doses taken in the morning and late afternoon/early evening. Following an additional 2–4 weeks, the dose may be further raised to a maximum of 100 mg for those who have not achieved an optimal response. It's worth noting that there is no evidence indicating that higher doses provide increased effectiveness.

In cases where strong CYP2D6 inhibitors are administered for children and adolescents over 70 kg, atomoxetine should also be started at 40 mg/day. The dosage can then be increased to the typical target dose of 80 mg/day only if symptoms do not improve after 4 weeks and the initial dose is well tolerated.

For ADHD patients with hepatic insufficiency (HI), dosage adjustments are necessary (Chalon et al., 2003). For those classified with moderate HI (Child-Pugh Class B), both the initial and target doses should be reduced to 50% of the standard dose. In cases of severe HI (Child-Pugh Class C), both the initial dose and target doses should be cut to 25% of the normal dosage.

However, administering the recommended starting dose of 0.5 mg/kg to children results in a 30-fold range in exposure, as indicated by dose-corrected AUC_0-∞_^19^, without considering the *CYP2D6* genotype or predicted phenotype. Simulated steady-state exposure profiles at the maximum recommended dose suggest that most children are unlikely to achieve adequate levels of atomoxetine exposure (Brown et al., 2016).

Given that a relatively small percentage of the population are CYP2D6 PMs [*e.g.*, around 7% in Caucasians (de Leon, 2015)], some experts argue that the currently approved clinical dosing may serve as a compromise for the majority of non-PMs. This is because the dosages are slightly lower to accommodate the potential tolerability or adverse reactions in PMs. However, this can lead to insufficient exposure among non-PMs, potentially affecting drug efficacy (Brown et al., 2016). Clearly, simply dose tailoring based solely on BW does not fulfill the need for personalized medication.

In our clinical practice, recent plasma atomoxetine monitoring revealed some intriguing trends: some children achieved higher levels of exposure at very low doses, while others had high doses but low systemic exposure. Meanwhile, some pediatric patients tolerated atomoxetine poorly with low exposure, while others managed well at high drug concentrations. In another scenario, some children on low doses experienced low exposures and tolerated the drug well, but demonstrated poor clinical efficacy. It is puzzling that the decision was made to not tailor the dosing, but instead to choose alternative medications (Fu et al., 2023).

## 3 Framework for assessing evidence backing personalized dosing of atomoxetine

Personalized prescribing for atomoxetine, like that for other medications, involves tailoring doses based on pharmacokinetic and pharmacodynamic mechanism that impact its safety and effectiveness. We utilize the Arbeitsgemeinschaft für Neuropsychopharmakologie und Pharmakopsychiatrie (AGNP) consensus guidelines to create a framework for evaluating the evidence supporting TDM of atomoxetine (Hiemke et al., 2018). This guideline offers recommendations regarding the timing of blood sampling after administration of atomoxetine, reference concentration ranges, and laboratory alert values; however, the evidence level provided is classified as Grade 3. In 2019, the Clinical Pharmacogenetic Implementation Consortium (CPIC) guidelines (Brown et al., 2019) estimated the activity score (AS) of CYP2D6 based on genotyping results, subsequently defining the phenotype of patients with these alleles. The guidelines made recommendations for the timing of blood sampling and the expected drug concentration range after atomoxetine use, with corresponding dose adjustments if the target concentration was not achieved.

We are particularly interested in exploring how these guidelines have contributed to advancing the practical implementation of personalized medication with atomoxetine and what new advancements have emerged in this field (Fu et al., 2023). To better organize information from previous reports, we modified the questions originally posed by Beumer et al. (2019) to focus on the clinical pharmacology of the medication, emphasizing key aspects relevant to TDM evaluation and personalized dosing.

## 4 Pharmacokinetics

### 4.1 Is there significant inter-individual variability in plasma concentrations using the current BW-based dosing regimen?

As shown in Table 3, the inter-individual variability in the plasma clearance of atomoxetine, corrected by oral bioavailability (CL/F), ranges between 14%–62% CV in children. This variability is closely associated with the CYP2D6 phenotype. Significant differences in plasma atomoxetine concentrations among children exist, depending on the route of administration (Guo et al., 2024). Most recently, Guo et al. (2024) identified sex, BW, and CYP2D6 phenotype were the primary factors influencing individual exposure to atomoxetine, with the phenotype exerting the most significant impact. Although the impact of CYP2D6 phenotype on pharmacokinetics of atomoxetine in children has been observed, studies in this area remains quite limited.

**TABLE 3:** Average values and inter-subject variability of atomoxetine exposure and clearance.

Dose/Regimen	Population					N	Parameter						Ref
	Genotype/Phenotype	Age (year)	Country/Race	Subgroup	Children/Adults		C_max_ (ng/mL)		AUC (ug/h/mL)		CL/F (L/h/kg)		
		Mean (SD)/Range					Mean (SD)	CV%	Mean (SD)	CV%	Mean (SD)	CV%	
10 mg/Single-Dose	CYP2D6 EM	10.9 (1.6)	America	-	Children	7	144 (53.4)	37.1	-	-	0.455 (0.160)	35	Witcher et al. (2003)
0.5 mg/kg/Single-Dose	CYP2D6 EM1^†^	9.5–17.8	Multiethnicity	-	Children	8	255.3	30	1.224	33.3	0.320	31.3	Brown et al. (2016)
	CYP2D6 EM2^†^			-		8	178.7	28.6	1.109	54.3	0.210	61.9	
	CYP2D6 IM			-		3	357.4	7.1	3.596	13.5	0.110	30	
	CYP2D6 p.m.			-		4	638.2	12	12.648	28.8	0.035	14.2	
20 mg/Single-Dose	-	52 (8)	America	Healthy control	Adults	10	142.2 (51.2)	36	0.690 (0.480)	69.1	0.506 (0.270)	53.5	Chalon et al. (2003)
	-	53 (9)		Child-Pugh B		6	115.8 (63.9)	55.2	1.160 (0.430)	37.3	0.208 (0.060)	28.1	
	-	55 (9)		Child-Pugh C		4	125.8 (56.4)	44.8	2.540 (1.430)	56.2	0.155 (0.120)	78.5	
40 mg/Single-Dose	CYP2D6 EM	20–39	China	-	Adults	16	449	32.1	3.630	47.6	0.241	62.6	Cui et al. (2007)
10 mg/Single-Dose	CYP2D6 EM	20–31	Japan	-	Adults	22	110.53	33.2	0.574	70.2	0.377	43.4	Matsui et al. (2012)
			America	-		16	84.54	37.4	0.512	69.7	0.356	47	
40 mg/Single-Dose			Japan	-		21	478.36	33.5	2.510	68.5	0.347	47.4	
			America	-		-	-	-	-	-	-	-	
90 mg/Single-Dose			Japan	-		20	920.03	33.1	5.300	54.2	0.337	40.1	
			America	-		15	812.55	30.2	5.47	30.2	0.289	41.5	
120 mg/Single-Dose			Japan	-		19	1,086.23	30.6	6.43	37.5	0.348	38.5	
			America	-		15	1,053.18	31.4	7.43	65.5	0.278	40.2	
40 mg/Single-Dose	-	19–29	China	Healthy male	Adults	22	437.82	37.6	2.693	98.8	-	-	Shang et al. (2013)
40 mg/Single-Dose	CYP2C19 EM	-	Korea	-	Adults	14	221.5	19.1	0.909	13	0.669	18.2	Choi et al. (2014)
	CYP2C19 IM	-		-		14	269.4	27.3	1.075	19	0.602	15.9	
	CYP2C19 p.m.	-		-		12	386.1	18.4	1.63	25.4	0.405	24	
40 mg/Single-Dose	CYP2D6*wt/*wt	23.1 (2.1)	Korea	-	Adults	22	340.1 (89.2)	2.6	1.254 (0.246)	19.6	0.824 (0.152)	18.5	Byeon et al. (2015)
12–40 mg/Single-Dose	CYP2D6*wt/*10	23.2 (2.3)		-		22	391.2 (105.6)	27.0	1.672 (0.363)	21.7	0.622 (0.127)	20.4	
	CYP2D6*10/*10	23.3 (2.9)		-		18	591.3 (144.2)	24.4	4.264 (1.190)	27.9	0.250 (0.061)	24.7	
25 mg/Single-Dose	-	18–55	Caucasian	ATX alone	Adults	20	226.43	42.4	1.583	65.7	-	-	Todor et al. (2017)
	-			ATX + FVX		20	283.09	36.7	2.111	66.9	-	-	
25 mg/Single-Dose	CYP2D6 EM	18–55	Caucasian	ATX alone	Adults	18	226	42.5	1.580	69	-	-	Todor et al. (2016)
				ATX + BUP		18	386	35.5	8.060	51.6	-	-	
	CYP2D6 p.m.			ATX alone		2	365	1.5	7.680	0.1	-	-	
				ATX + BUP		2	377	1.1	9.750	1.7	-	-	
20 mg/Steady-State	-	20–49	America	ATX alone	Adults	21	184	36	0.846	45	0.395	55	Belle et al. (2002)
	-		America	ATX + PRX		14	690	37	5.970	42	0.060	81	
20 mg/Steady-State	CYP2D6 EM	38–54	America	-	Adults	4	159.7 (82.9)	51.9	1.080 (0.690)	64.3	0.373 (0.280)	75.1	Sauer et al. (2003)
	CYP2D6 p.m.	19–49	America	-		3	914.72 (279)	30.5	8.44 (2.27)	26.9	0.0357 (0.0093)	26.2	
20–45 mg/Steady-State	CYP2D6 EM	10.9 (1.6)	America	-	Children	7	537 (306.1)	57	-	-	0.455 (0.160)	35	Witcher et al. (2003)
40 mg/Steady-State	-	38–54	America	ATX alone	Adults	6	552.41	45	3.180	84.6	0.327	73	Sauer et al. (2004)
	-		America	ATX + DMI		6	556.73	47.8	3.470	76.3	0.27	66.6	
60 mg/Steady-State	-		America	ATX alone		15	590.81	46.3	2.690	56.6	0.399	62	
	-		America	ATX + DMI		15	646.63	34.5	3.010	51.5	0.343	58.3	
80 mg/Steady-State	CYP2D6 EM	-	Korea	-	Adults	16	1,020	32.7	7.120	48.2	0.242	58.7	Choi et al. (2014)
Initiated at 0.5 mg/kg/day and increased to 1.2 (max to 1.8) mg/kg/day	-	11.6 (2.4) 11.2 (2.7)	America	ATX alone	Children	46	351.0	105	-	-	-	-	Kratochvil et al. (2005)
				ATX + FLX		127	1,176.7	48	-	-	-	-	

Notes: EM, IM, and PM, refer to extensive metabolizer, intermediate metabolizer and poo metabolizer, respectively.

^†^ EM1 defined as extensive metabolizers with one functional and one nonfunctional allele, or two reduced function alleles and EM2 defined as extensive metabolizers with two or more functional alleles. ATX, FVX, BUP, PRX, DMI, and FLX, are abbreviated to atomoxetine, fluvoxamine, bupropion, paroxetine, desipramine, and fluoxetine, respectively.

### 4.2 Is there limited intra-individual variability in plasma concentrations?

Currently, reports on the intra-individual differences in pharmacokinetic parameters, such as plasma atomoxetine concentrations and total CL, are very limited. Recently, Cheng et al. (2023) utilized a population pharmacokinetic (PPK) modeling approach to estimate the residual unexplained variability (i.e., intra-individual variability) in plasma concentrations of atomoxetine and its major metabolite, 4-OH-atomoxetine, in children and adolescents, yielding 21.3% and 29.6% CV, respectively. In addition, early studies have indicated that, food intake reduces its peak concentration and delays the time to peak concentration, although it does not affect the absorption of atomoxetine (Sauer et al., 2005). This factor should also be considered as contributing to both intra-individual and inter-individual variability.

### 4.3 Do drug-drug interactions (DDIs) impact the pharmacokinetic parameters of atomoxetine?

When ADHD occurs alongside other conditions such as anxiety or depression, there may be a need for using atomoxetine in combination with other medications; however, this is more common in adults (Todor et al., 2017; Todor et al., 2016; Belle et al., 2002; Sauer et al., 2004; Kratochvil et al., 2005). Research has evaluated the impact of bupropion, fluvoxamine, paroxetine, desipramine, and fluoxetine on the pharmacokinetics of atomoxetine, as a victim drug (Table 3). Studies involving fluvoxamine (Todor et al., 2017) and desipramine (Sauer et al., 2004) indicated that interactions, if present, were slight; however, fluoxetine increased atomoxetine peak concentration by 3.4 fold (Kratochvil et al., 2005). Of note, bupropion exhibited significant inhibitory effects on atomoxetine’s metabolism in patients classified as CYP2D6 EMs, while the effects were minimal in CYP2D6 PMs (Todor et al., 2016). Interestingly, a Canadian guideline classifies bupropion as a third-line treatment for ADHD (Schoretsanitis et al., 2019). In addition, a case report has noted an improved response to atomoxetine in a patient, likely classified as a CYP2D6 EM, following the addition of paroxetine (Paulzen et al., 2016), indicating that these inhibitors can be utilized to enhance the response to atomoxetine in individuals identified as CYP2D6 EMs.

Conversely, a recent study involving children and adolescent with ADHD revealed that the use of concomitant medications is quite rare, particularly regarding herbal medicines (Guo et al., 2024). Nevertheless, instances in adults where the concurrent use of medications as potential inhibitors of CYP2D6 (Table 4) significantly alters the systemic exposure to atomoxetine warrant careful consideration by pediatricians. If similar combinations of medications become necessary for children and adolescents, it may be important to adjust doses to account for changes in exposure due to DDIs (Sauer et al., 2004) in order to ensure efficacy and minimize adverse reactions (Fu et al., 2023).

**TABLE 4:** FDA examples of clinical inhibitors for CYP2D6 (Cicali et al., 2020).

FDA classification	Medication
Strong inhibitor	quinidine, paroxetine, fluoxetine, bupropion
Moderate inhibitor	cimetidine, cinacalcet, duloxetine, fluvoxamine, mirabegron
Weak inhibitor	abiraterone, amiodarone, celecoxib, cimetidine, clobazam, cobicistat, desvenlafaxine, escitalopram, labetalol, lorcaserin, ritonavir, sertraline, vemurafenib

It is crucial to highlight that whether CYP2D6 inhibitors have a practical effect is closely linked to the patient’s CYP2D6 metabolic phenotype. They do not work in CYP2D6 PMs but can enhance systemic exposure to atomoxetine in CYP2D6 non-PMs. Therefore, gathering genotype and phenotype information about the patient’s CYP2D6 status becomes necessary.

## 5 Pharmacodynamics (PD)

### 5.1 Is there a narrow therapeutic window?

As of now, the therapeutic window for atomoxetine in treating ADHD in children has not been clearly established. The recommended concentration range of 200–1,000 ng/mL, as outlined in guidelines (Hiemke et al., 2018; Brown et al., 2019), primarily focuses on identifying the lowest concentration necessary to achieve efficacy. However, the link between plasma levels of atomoxetine and its clinical effectiveness remains unclear, complicating the relationship between tolerability and concentration even further (Guo et al., 2024). Consequently, it is more plausible that we will first define a minimum concentration required for therapeutic effect, while finding a corresponding concentration that ensures tolerability is challenging, as tolerability does not always have a straightforward relationship with drug concentration.

### 5.2 Are there easy and clinically relevant biomarkers to predict response and/or toxicity at a given dose?

#### 5.2.1 CYP2D6

Atomoxetine is primarily cleared from the body through oxidative metabolism, with the majority of its oxidative metabolites being excreted in the urine. This metabolic process is predominantly facilitated by CYP2D6, making the polymorphism of CYP2D6 significantly relevant to the pharmacokinetics of atomoxetine (Sauer et al., 2005). CYP2D6 affects both the efficacy and tolerability of atomoxetine by influencing its pharmacokinetic processes in the body (Guo et al., 2024), positioning it as a crucial biomarker to monitor and investigate at this time (Nijenhuis et al., 2023; Brown, 2022; Michelson et al., 2007).

To date, over 160 star alleles of *CYP2D6* have been cataloged in the PharmGKB and CPIC databases. Each star allele can exhibit varying levels of activity, which may clinically manifest as normal function, increased function, decreased function, or no function at all. It is important to highlight that the functionality of over half of the alleles remains uncertain or unknown, leading to ambiguity or complexity regarding the ability of individuals with these alleles to metabolize atomoxetine (Table 5).

**TABLE 5:** *CYP2D6* genotype^†^.

Allele clinical functional status	Alleles	Activity values
Normal function	*1, *2, *17 × 2, *27, *29 × 2, *33, *34, *35, *39, *45, *46, *48, *53	1
Increased function	*1 × 2, *1x ≥ 3, *2 × 2, *2x ≥ 3, *35 × 2, *45 × 2	2, ≥3.0, 2, ≥3.0, 2, 2
Decreased function	*9, *9 × 2, *10, *10 × 2, *14, *17, *29, *32, *41, *41 × 2, *41 × 3, *49, *50, *52, *54, *55, *59, *91, *109, *119, *132	0.25, 0.5, 0.25, 0.5, 0.5, 0.5, 0.5, 0.25, 0.25, 0.5, 0.75, 0.5, 0.5, 0.25, 0.5, 0.5, 0.5, 0.25, 0.25, 0.25, 0.25
No function	*3, *3 × 2, *4, *4 × 2, *4x ≥ 3, *5, *6, *6 × 2, *7, *8, *11, *12, *13, *15, *18, *19, *20, *21, *31, *36, *36 × 2, *38, *40, *42, *44, *47, *51, *56, *60, *62, *68, *69, *81, *92, *96, *99, *100, *101, *114, *120, *124, *129, *143, *144, *156, *161	0
Uncertain function	*22, *23, *24, *25, *26, *28, *30, *37, *43, *43 × 2, *61, *63, *64, *65, *70, *71, *72, *75, *83, *84, *87, *88, *89, *90, *93, *94, *95, *97, *98, *106, *110, *111, *112, *113, *123, *128, *130, *131, *133, *134, *135, *136, *137, *138, *141, *142, *145, *146, *146 × 2, *147, *154, *162	n/a
Unknown function	*58, *73, *74, *82, *85, *86, *102, *103, *104, *105, *107, *108, *115, *116, *117, *118, *121, *122, *125, *126, *127, *139, *140, *148, *149, *152, *153, *155, *157, *158, *159, *160, *163	n/a

Notes: ^†^ This table was modified according to the Gene-specific Information Tables for CYP2D6 (https://www.pharmgkb.org/page/cyp2d6RefMaterials; Access time, 2024/4/4).

While there are over 14,700 possible combinations of CYP2D6 diplotype (CYP2D6 Diplotype-Phenotype Table, https://www.pharmgkb.org/page/cyp2d6RefMaterials; last accessed, 2024/4/4), they can be generally categorized into the following phenotypes based on AS: ultrarapid metabolizer (UM), normal metabolizer (NM; formerly extensive metabolizers, EM (Nofziger et al., 2020)), intermediate metabolizer (IM), and PM. The prevalence of these phenotypes varied significantly across biogeographical groups, with the majority of populations classified as NM and IM, whereas UM and PM phenotypes are less frequently observed (Table 6).

**TABLE 6:** CYP2D6 phenotype and frequencies^†^.

Phenotype	Activity score	Activity value allele 1	Activity value allele 2	Frequencies of CYP2D6 phenotypes in biogeographical groups (%)								
				African American/Afro-Caribbean	American	Central/South asian	East asian	European	Latino	Near eastern	Oceanian	Sub-Saharan african
Ultrarapid Metabolizer (UM)	≥6.0	≥3.0	≥3.0	4.08	5.14	1.50	0.86	2.33	4.07	7.44	17.8	3.60
	≥5.0	≥3.0	2									
	≥4.0	≥3.0	1									
	4	2	2									
	≥3.75	≥3.0	0.75									
	≥3.25	≥3.0	0.25									
	≥3.5	≥3.0	0.5									
	≥3.0	≥3.0	0									
	3	2	1									
	2.75	2	0.75									
	2.5	2	0.5									
Normal Metabolizer (NM)	2.25	2	0.25	53.8	64.9	58.1	53.8	49.2	59.6	56.5	63.6	25.4
	2	2	0									
	2	1	1									
	1.75	0.75	1									
	1.25	0.75	0.5									
	1.25	1	0.25									
	1.5	0.75	0.75									
	1.5	1	0.5									
Intermediate Metabolizer (IM)	1	0.75	0.25	35.9	23.1	28.1	38.3	38.3	29.1	30.1	9.5	33.9
	1	1	0									
	1	0.5	0.5									
	0.75	0.75	0									
	0.75	0.5	0.25									
	0.25	0.25	0									
	0.5	0.5	0									
	0.5	0.25	0.25									
Poor Metabolizer (PM)	0	0	0	2.35	2.02	2.35	0.79	6.50	3.12	2.20	0.31	2.04
CYP2D6 Indeterminate^b^	n/a			3.89	4.92	9.99	6.27	3.73	4.16	3.74	8.73	35.0

Notes: ^†^This table was modified according to the Gene-specific Information Tables for CYP2D6 (https://www.pharmgkb.org/page/cyp2d6RefMaterials; Access time, 2024/4/4); ^‡^An individual carrying one allele with one known function allele (like increased, decreased, normal, or no function allele) and one uncertain or unknown function allele.

In Oceania, the frequency of UMs is nearly 20%, indicating that patients with enhanced function alleles may experience very low systemic exposure levels from the same dose of atomoxetine, which could lead to poor efficacy. Conversely, the frequency of UMs in East Asia is below 1%, making the likelihood of this scenario one-twentieth that of Oceania. Furthermore, PMs have the lowest distribution frequencies in both Oceania and East Asia, at 0.31% and 0.79% respectively. This suggests that the risk of excessive atomoxetine exposure due to non-functional metabolizing enzymes is relatively low, implying a potentially reduced likelihood of poor tolerability in these populations compared to others (Table 6).

For example, the oral clearance in CYP2D6 PMs was only 6.0% of that observed in the EM2 group, potentially resulting in higher exposure to atomoxetine. In PMs, the half-life (*t*_1/2_) was 2.9 times longer than t in the IMs, and 5.4 to 5.9 times longer than in both EM1 and EM2 groups, with the AUC _0-∞_ showing a variability of 29.6 times across the study cohort (Brown et al., 2016). Similarly, at comparable doses for children and adolescents with ADHD, the mean peak atomoxetine concentrations in CYP2D6 PMs were approximately 5 times higher than those in EMs (Michelson et al., 2007).

In a study examining the correlation between pharmacogenetics and treatment response, 589 participants—30 CYP2D6 PMs and 559 CYP2D6 EMs—completed a treatment period lasting 6–8 weeks, during which their responses were evaluated. The average improvements, assessed using the ADHDRS IV Parent Interview, were 14.1 points for EMs and 20.9 points for PMs. The response rates, defined as a 25% decrease from baseline in ADHDRS-IV-Parent: Inv total score at study endpoint, were 59.4% for EMs and 80% for PMs, respectively (Michelson et al., 2007).

In a group of 100 children, Ter Laak et al. (2010) identified 10 candidates for *CYP2D6* genotyping due to delayed response or poor tolerability. Among these, 8 children were found to be CYP2D6 PMs; 4 experienced improved therapeutic effects after dose reduction, while the remaining 4 discontinued treatment due to initial adverse reactions. As a result, the authors suggested that pre-emptive genotyping for *CYP2D6* could enhance the efficacy of atomoxetine and help manage its adverse effects. Additionally, cases with rs1135840 (Chatterjee et al., 2023) (4180 G>C, decreased function) “CC” showed improvement after atomoxetine treatment. However, some other studies indicated that routine genotyping might not be necessary, as researchers managed to dose atomoxetine effectively, achieving similar efficacy and safety levels in both EMs (n = 1,239) and PMs (n = 87) without prior knowledge of their metabolizer status (Trzepacz et al., 2008). Nevertheless, the clinical characteristics of PMs prompted healthcare providers to consider reducing dosages for these individuals, even without information about their metabolic status.

Selecting the appropriate clinical *CYP2D6* genotyping alleles is crucial for standardizing gene testing across clinical labs. Recently, several organizations, including the Association for Molecular Pathology, College of American Pathologists, Dutch Pharmacogenetics Working Group of the Royal Dutch Pharmacists Association, and the European Society for Pharmacogenomics and Personalized Therapy, released a Joint consensus recommendation regarding the selection of these alleles (Pratt et al., 2021). This guidance outlines a foundational set of variant alleles (Tier 1) and an expanded set (Tier 2) to aid clinical labs in developing *CYP2D6* testing assays. Briefly, the Tier 1 recommended *CYP2D6* variant star alleles include *2 through *6, *9, *10, *17, *29, and *41, along with the determination of gene duplication or multiplication status. The Tier 2 recommended *CYP2D6* variant alleles consist of *7, *8, *12, *14, *15, *21, *31, *40, *42, *49, *56, and *59, as well as hybrid genes that contain segments of both *CYP2D6* and *CYP2D7*.

The next consideration is how to achieve rapid and cost-effective genotyping. In clinical labs, various methods (Table 7) for detecting CYP2D6 haplotypes are already being used, including techniques capable of identifying hybrid arrangements and quantify copy number variants (CNVs). Long-range polymerase chain reaction (PCR) or extra-long-range PCR methods are designed to amplify the entire *CYP2D6* gene, allowing for the detection of multiple copies or whole-gene deletions. While these methods are robust and reliable, they can be time-consuming and may not be suitable for the rapid screening of a wide range of alleles (Taylor et al., 2020). In addition, long range-PCR followed by Sanger sequence is considered the gold-standard for definitive *CYP2D6* genotype determination when CNVs are present; however, this approach is labor-intensive and involves complex procedures (Atiq et al., 2023).

**TABLE 7:** Detection approaches for CYP2D6 genotyping.

Methods	Sample	Advantages	Disadvantages	References
Long-range polymerase chain reaction (PCR) or extra-long-range PCR	Whole blood	Robust and reliable	Time-consuming and not suited to the rapid screening of a large number of different alleles. It is mainly suitable in a clinical setting where the allelic variants of the screened population are predictable	Taylor et al. (2020)
Pyrosequencing	Whole blood	An inexpensive high-throughput sequencing method in comparison to traditional Sanger sequencing	It is challenging to interpret and require additional instrumentation and an additional workflow to implement	Siqueira et al. (2012)
Long range-PCR couple with Sanger sequence	Whole blood	High accuracy, a gold-standard for definitive CYP2D6 genotype determination when copy number variants (CNVs) are present	Weak ability to identify novel variants, relative lower throughput. Labor-intensive, requires additional reagents and set-ups	Atiq et al. (2023)
TaqMan assays	Whole blood	High specificity and accurate quantification, short experimental time	High cost, difficult probe design	Mbavha et al. (2023)
High Resolution Melt analysis	Whole blood	Simple assay, high sensitivity, high throughput, and low cost	With false positives risk, and need high technical requirements for detection personnel	Moric-Janiszewska et al. (2023)
AmpliChip CYP450 GeneChip	Whole blood	Allowing a fast, accurate and comprehensive identification of CYP2D6 genotypes	Relatively high costs	Heller et al. (2006)
GenoChip CYP2D6 macroarray	Whole blood	Low in costs and easy to handle	In individuals who are carriers of a variant allele and a duplication of an allele, the interpretation of the results of the GenoChip CYP2D6 can lead to multiple diplotypes	Bank et al. (2015)
Stargazer	Algorithms infer CYP2D6 haplocyte from next-generation sequencing (NGS) data	Stargazer is the only tool that uses statistical haplotype phasing, which is informed by population haplotype frequencies to call star alleles more accurately	Aldy and Stargazer rely on accurate read alignments, which may not be possible at many positions throughout the gene as the sequence is highly similar or even indistinguishable with CYP2D7	Lee et al. (2019), Rosenbaum (2020)
Aldy		Aldy is able to identify a large set of hybrid/fusion genes, composed of a coding gene and a highly similar pseudogene; with minimal impact on computational resources		Numanagic et al. (2018)
Constellation		Rapid, scalable and has minimal incremental cost in the setting of NGS.	Cypiripi and Constellation were not designed to detect complex SVs and have been shown to have relative lower performance	Twist et al. (2016)
Cypiripi		With highly optimized running time, and can be easily extended to other unique gene clusters with similar properties		Numanagic et al. (2015)
Cyrius		Overcomes the challenges with the homology between CYP2D6 and CYP2D7, and with a higher accuracy (96.5%)	There is no truth data available to validate the remaining, rarer star alleles defined by PharmVar	Chen et al. (2021)

Pyrosequencing is an cost-effective high-throughput sequencing method compared to traditional Sanger sequencing, although it presents challenges in interpretation and requires additional instrumentation and workflows to for implementation (Siqueira et al., 2012). The Taqman assay offers an alternative method, using bioluminescent tagged probes that provide high specificity and accurate quantification, along with short experimental duration, albeit with challenges in probe design (Mbavha et al., 2023). Additionally, there are specialized commercial products, such as the AmpliChip CYP450 assay and GenoChip *CYP2D6* macroarray, which are effective tools for *CYP2D6* genotypes (Heller et al., 2006; Bank et al., 2015). These methods provide an efficient and rapid means of advancing the application of pharmacogenetics in clinical settings.

Recent advances in next-generation sequencing (NGS) have led to the development of several algorithms for inferring *CYP2D6* haplotype from NGS data, including Stargazer, Aldy, Constellation, Cypiripi and Cyrius (Lee et al., 2019; Rosenbaum, 2020; Numanagic et al., 2018; Twist et al., 2016; Chen et al., 2021). These approaches provide a valuable way for predicting an individual’s metabolism, making the use existing data more cost-effective and widely accessible.

However, there are various reasons why genotyping for *CYP2D6* may not be feasible (Brown et al., 2021; Chenoweth et al., 2020). In such cases, alternative methods for sequencing the *CYP2D6* gene become particularly important. For example, Shimizu et al. revealed that utilizing AUC values for average daily urinary excretion could be an effective way to estimate the CYP2D6 phenotype in pediatric patients (Shimizu et al., 2023). Additionally, the relatively narrow ranges of 4-hydroxyatomoxetine and N-desmethyl-atomoxetine concentration ratios in spot urine samples from children could serve as a simple, semi-quantitative indicator of CYP2D6 IMs (Shimizu et al., 2023).

By obtaining the AS of CYP2D6 through methods other than genotyping, it becomes possible to predict the pharmacokinetic parameters of atomoxetine. For example, physiologically based pharmacokinetic (PBPK) models have been successfully used to describe and predict the AS-dependent metabolism of CYP2D6 substrates like atomoxetine based on plasma concentration-time profiles. In the absence of *CYP2D6* genotype data, plasma atomoxetine concentrations have been successfully predicted using generally known AS values (Rudesheim et al., 2022). Furthermore, Cheng et al. (2023) developed an comprehensive PPK model to describe the pharmacokinetic profiles of atomoxetine and its metabolites in both plasma and urine, incorporating the effects of CYP2D6’ ASs and BW on model parameters, which is anticipated to aid in future optimization of atomoxetine dosing.

#### 5.2.2 Other potential biomarkers

Based on the mechanism of action of atomoxetine (Figure 1), some studies have explored the potential of other substances as neurodevelopmental biomarkers. Examples include 3,4-dihydroxy phenylethylene glycol (DHPG) (Kielbasa and Lobo, 2015; Kielbasa et al., 2015; Bieck et al., 2016; Montoya et al., 2011), dopamine β-hydroxylase (DBH) (Fang et al., 2015), norepinephrine transporter (NET) (Chatterjee et al., 2023; Gul et al., 2022; Yang et al., 2013), and Brain-derived neurotrophic factor (BDNF) (Demirci et al., 2022; Ramos-Quiroga et al., 2014). In addition, various plasma and urinary metabolites from children with ADHD have also been identified, which may serve as potential markers for further study (Wang et al., 2021; Tian et al., 2022).

## 6 Exposure-response (PK-PD)

### 6.1 Is there an accepted and clinically relevant metric for systemic exposure to atomoxetine?

Atomoxetine is taken orally, and main pharmacokinetic parameters identified in the literature include plasma peak concentration (*C*_*max*_), AUC, and CL/F (Table 3), as along with plasma concentrations measured at specific intervals after administration (*e.g.*, 12 h). Current clinical evidence strongly associates *C*_*max*_ with the efficacy of atomoxetine, which is why it is recommended as a primary monitoring parameter in guidelines (Hiemke et al., 2018; Brown et al., 2019). Although establishing a connection between *C*_*max*_ and adverse reactions can be more difficult, some findings in the literature address this relationship as well (Guo et al., 2024).

### 6.2 Is there evidence for the relationship between plasma atomoxetine concentration and clinical activity?

A systematic review and dose-response meta-analysis found that the effectiveness of atomoxetine increased up to a dosage of 1.4 mg/kg, after which it plateaued (Terao et al., 2024). There is considerable interest in determining if specific plasma concentrations of atomoxetine can predict the level of clinical response. In an early investigation, Michelson and co-researchers applied a nonlinear model to analyze peak concentrations and the relative change from baseline in the ADHDRS-IV-Parent: Inv total score. This model indicated that the maximum expected improvement compared to baseline would be −23.5, aligning with a plasma atomoxetine concentration of 400 ng/mL (Michelson et al., 2007). However, Hazell et al. revealed that while certain patients may benefit from higher plasma atomoxetine levels (>800 ng/mL), mere exposure to these levels dose not reliably predict the therapeutic outcomes in children with ADHD, suggesting that other factors also influence the response to atomoxetine (Hazell et al., 2009).

In a recent non-randomized prospective interventional study, Sugimoto et al. (2021) found that children with ADHD aged 6–12 years (n = 43) were more likely to respond to respond to atomoxetine treatment when its steady-state plasma concentration exceeded 64.60 ng/mL. Similarly, Guo et al. (2024) identified a lower threshold of 268 ng/mL as a potential therapeutic reference range for pediatric patients receiving *q.m.* atomoxetine, suggesting that effectiveness increases when this level is surpassed. Conversely, Ruppert et al. (2022) found that neither a concentration-effect relationship nor a dose-effect relationship was observed.

### 6.3 Is there evidence for the relationship between plasma atomoxetine concentration and tolerability?

In general, CYP2D6 PMs are more likely to experience side effects from atomoxetine than non-PMs, likely due to their higher exposure to the drug (Brown et al., 2019; Michelson et al., 2007). However, it is still uncertain whether drug exposure metrics like *C*_max_ or AUC have a significant influence on tolerability, as there are only a few studies investigating the relationship between plasma atomoxetine concentrations and clinical outcomes. One early clinical trial found no correlation between plasma atomoxetine concentrations and its tolerability (Hazell et al., 2009). Similarly, a TDM study involving children and adolescents with ADHD did not reveal any clear relationship between serum concentrations and side effects (Ruppert et al., 2022). In contrast, a recent study by Guo et al. (2024) did identify a correlation between certain adverse reactions and plasma atomoxetine concentration. Specifically, in CYP2D6 IMs receiving once-daily dosing or EMs receiving twice-daily dosing, a significant difference was observed in the occurrence of gastrointestinal (*e.g.*, 510 vs. 386 ng/mL, *p* = 0.0411) and neurological adverse reactions, even at plasma atomoxetine concentrations where no adverse reactions were reported.

## 7 Evaluation of TDM

TDM involves measuring and interpreting drug concentrations in biological fluids such as plasma and serum to tailor drug dosages or schedules, maximizing therapeutic benefits while minimizing toxicity for individual patients. Since 2000, the AGNP TDM guidelines have offered valuable direction for adjusting dosages of various psychiatric medications (Hiemke et al., 2018; Baumann et al., 2004; Ulrich et al., 2007; Schafer et al., 2016; Hiemke, 2016). The AGNP TDM guidelines in neuropsychopharmacology, established in 2011, along with the 2019 CPIC guidelines (Brown et al., 2019), both recommend TDM for atomoxetine. These guidelines may have played a crucial role in promoting the appropriate use of atomoxetine for patients with ADHD.

### 7.1 Is there evidence that TDM improves effectiveness in patients receiving atomoxetine?

As of now, no studies have directly compared the therapeutic effects of atomoxetine before and after the implementation of TDM. However, clinicians at our hospital believe that TDM has significantly enhanced their ability to select medications and adjust dosages. With TDM support, they can make more timely clinical decisions, such as switching from atomoxetine to alternative medications or tailoring the dosage. This approach has allowed for a more efficient determination of the optimal dosage for pediatric patients using atomoxetine. Notably, some children with ADHD have experienced effective control with relatively lower doses, an outcome that was less common prior to TDM implementation. We are currently gathering such real-world clinical data and planning to design clinical trials to systematically evaluate the benefits of implementing TDM for atomoxetine.

### 7.2 Is there evidence that TDM reduces tolerability in patients receiving atomoxetine?

Toxicity from overdose is thought to arise from elevated synaptic NE levels, which can induce an excessive noradrenergic-mediated sympathomimetic syndrome, which typically presents as tachycardia and hypertension. In cases of atomoxetine overdose, clinical manifestations are generally mild. Common symptoms include drowsiness (particularly in children), agitation, hyperactivity, gastrointestinal disturbances, tremors, hyperreflexia, tachycardia, hypertension, and seizures. Fortunately, these symptoms typically resolve quickly, with complete recovery usually occurring within 24 h post-overdose (Spiller et al., 2013). However, some patients may need to discontinue atomoxetine due to inability to tolerate several common adverse reactions reported in clinical trials, including nausea, vomiting, fatigue, decreased appetite, abdominal pain, and somnolence (Mechler et al., 2022).

Similarly, there has been no public report to date examining whether the tolerability of atomoxetine improves before and after the implementation of TDM. Additionally, the correlation between tolerability and concentration has also not been established. However, it is indeed more likely for PMs to experience adverse reactions compared to non-PMs (Brown et al., 2019). From this perspective, implementing TDM is expected to improve tolerability. For example, TDM could help identify children on low doses of atomoxetine who have low exposure, resulting in poor tolerance and inadequate efficacy. In such cases, a timely medication switch may be appropriate, potentially avoiding the need for further dose escalation to achieve efficacy. Conversely, for children with high exposure who show good efficacy and good tolerance, a dose reduction can be considered to alleviate the body’s burden of atomoxetine. This area warrants exploration in clinical trial and represents a significant clinical issue that should be prioritized.

## 8 Clinical implementation

### 8.1 Are reliable assays available?

Various bioanalytical assays have been established to measure atomoxetine in human plasma, serum, urine, or hair, using techniques such as (high-performance) liquid chromatography combined with detection methods such as UV detector (Patel et al., 2007; Guo et al., 2007; Teichert et al., 2020), fluorescence detector (Stegmann et al., 2016; Zhu et al., 2007), or by (tandem) mass spectrometry (Mullen et al., 2005; Papaseit et al., 2012; Papaseit et al., 2013; Sim et al., 2017; Choi et al., 2012; Marchei et al., 2012; Xia et al., 2021; Skaalvik et al., 2021). Most of these methods are lab-developed and may have limited general applicability. Recently, new strategies have emerged recently. For example, Abu-Hassan developed a Nano-level assay based on molecular-size-based resonance Rayleigh scattering to detect atomoxetine in both its prescribed dosage form and plasma samples. This environmentally friendly fluorometric technique shows considerable promise for application due to its significant advantages, such as intelligent selectivity, exceptional sensitivity, minimal solvent consumption, widespread availability in laboratories, rapid analysis times, and ease of use (Abu-Hassan, 2023). Importantly, the choice of method is less critical than ensuring accurate determination of atomoxetine concentration in biological samples; researchers can select an assay based on its accessibility.

### 8.2 Is the proper sampling timing and handling established?

When performing TDM for atomoxetine, several factors need to be considered regarding blood collection methods. First, the genetic polymorphism of *CYP2D6* and metabolic phenotypes (UM, NM, IM, and PM) of CYP2D6 significantly influence the drug’s metabolism, leading to significant differences in its *t*_1/2_. Notably, the *t*_1/2_ of atomoxetine in PMs was 4-fold higher than that of EMs (Sauer et al., 2005; Michelson et al., 2007; Byeon et al., 2015). In clinical practice, *C*_max_ is primarily used as a parameter to assess the correlation between systemic exposure to atomoxetine, its effectiveness, and adverse reactions. Consequently, patients with different CYP2D6 phenotypes may experience varying peak times even under the same dosing regimen. Given the pharmacokinetic variations linked to CYP2D6 phenotypes that affect *C*_max_ and *t*_1/2_, the CPIC guideline recommends that prescribers consider measuring peak concentrations at specific time intervals: 1) 1–2 h post-dose in known CYP2D6 UMs, NMs, and IMs with high activity (AS 1.0 without the CYP2D6*10 allele); 2) 2–4 h post-dose in CYP2D6 IMs with low activity (AS 0.5) and individuals with an AS of 1 who carry the CYP2D6*10 allele; and 3) 4 h post-dose for PMs (Brown et al., 2019).

Second, the dosing regimen also affects the timing blood sample collection. For *q.m.* and *b.i.d.* regimens, it's generally straightforward to collect blood samples 1–4 h after drug administration. However, for children who take medication once at night (*q.n.*; not many, but seen (Mechler et al., 2022)), determining the interval (*e.g.*, 12 h) (Guo et al., 2024; Sugimoto et al., 2021) for blood sample collection can be a challenging issue. This situation may necessitate prior communication with the physician regarding the timing of the previous night’s medication and the blood collection time the following day.

Third, if genetic and phenotypic information is not available, or even if it is, a concentration obtained from a single time-point sample may not accurately represent the *C*_max_. The CPIC guideline also suggest collecting blood sample within a specific time window after dosing (Brown et al., 2019). Therefore, it may be necessary to consider sampling at steady state, despite studies that investigate the relationship between steady-state trough concentrations and clinical response (Sugimoto et al., 2021).

### 8.3 Is there a recommended therapeutic exposure range based on the clinical evidence?

As of now, two guidelines provide recommendations for the therapeutic reference range of atomoxetine. According to the AGNP TDM Expert Group consensus guidelines, peak plasma concentrations between 200 and 1,000 ng/mL, measured 60–90 min after a dose of 1.2 mg/kg/day, are commonly regarded as the therapeutic reference range, but this has only been studied in adults (Hiemke et al., 2018). The latest CPIC guideline also establishes a therapeutic reference range for peak plasma concentration at 200 and 1,000 ng/mL, noting that adequate responses can be achieved when the plasma concentrations exceed 400 ng/mL (Brown et al., 2019).

Interestingly, a recent retrospective study by Guo et al. involving children with ADHD, recommended a minimum *C*_max_ of 268 ng/mL associated with achieving a favorable therapeutic effect for patients receiving *q.m.* dosing of atomoxetine (Guo et al., 2024). Additionally, a naturalistic study in children and adolescents with ADHD proposed a therapeutic reference range of 100–400 ng/mL (Sugimoto et al., 2021). Researchers also recommend the minimum steady-state trough concentration of 64.6 mg/mL necessary for a good control of ADHD symptoms (Sugimoto et al., 2021).

### 8.4 Is there a dose-adaptation strategy?

In 2019, the CPIC released guidelines proposing the use of plasma concentration in conjunction with an individual’s *CYP2D6* genotype to assist clinicians in dose selection and titration. For patients classified as CYP2D6 UMs and NMs, if the peak concentration is <200 ng/mL and there is no clinical response, it is advisable to increase the dose proportionately to achieve approximately 400 ng/mL. For CYP2D6 PMs, IMs, and NMs with an AS of 1 who carry the CYP2D6*10 allele and taking a standard starting dose, the recommendation is to consider a proportional dose adjustment to reach about 400 ng/mL if there is an inadequate response without side effects (Brown et al., 2019).

In recent years, there have been extensive efforts to create personalized dosing strategies for atomoxetine using PBPK (Shimizu et al., 2023; Rudesheim et al., 2022; Dinh et al., 2016; Kim et al., 2018; Notsu et al., 2020; Alsmadi et al., 2022) and PPK (Cheng et al., 2023) models. Of note, recent PPK simulations revealed that the majority of individuals with a CYP2D6 AS of 1–3 may not achieve a steady-state *C*_max_ of 400 ng/mL with a 0.5 mg/kg once daily (*q.d.*) dosage, whereas most individuals with a CYP2D6 AS<1 could reach this concentration. This suggests that individuals with CYP2D6 AS of 1–3 may require a higher dose of atomoxetine compared to those with scores <1. To achieve a steady-state atomoxetine *C*_max_ comparable to that of individuals with an AS of 0 following a 0.5 mg/kg *q.d.* dose of atomoxetine, individuals with CYP2D6 AS 1-3 would require an approximately 1.2 mg/kg *q.d.* dose (Cheng et al., 2023). These findings largely align with the dosing recommendations outlined in the above noted CPIC guideline (Brown et al., 2019).

## 9 Cost effectiveness analysis of TDM and genotyping testing

### 9.1 Is there a cost effectiveness analysis of TDM testing for atomoxetine?

Cost-effectiveness analysis in healthcare, particularly for TDM, is still developing. Initially, TDM was only shown to be cost-effective for aminoglycosides (Touw et al., 2005). However, recent evidence indicates that TDM interventions can also be cost-effective in the application of antibody drugs (Martelli et al., 2017) and anti-cancer medications (Vithanachchi et al., 2021). While there is some rationale supporting the TDM of atomoxetine, comprehensive cost-effectiveness analyses have not yet been conducted. Consequently, the emphasis should extend beyond just cost-effectiveness to encompass how these interventions can be implemented in a clinically beneficial and economically sustainable way.

### 9.2 Is there a cost effectiveness analysis of *CYP2D6* genotyping testing for atomoxetine?

When integrating pharmacogenomics (PGx) into clinical practice, cost is also a crucial consideration for both healthcare systems and patients (Morris et al., 2022). Despite a substantial decrease in of PGx testing costs over the past decade, it continues to pose a significant barrier to widespread implementation in children’s hospitals (Brown et al., 2021). For certain medications, such as clopidogrel and warfarin, there is considerable cost data available that provide strong support for the use of PGx testing (Dong et al., 2020; Zhu et al., 2021). Additionally, cost-effectiveness analyses of *CYP2D6* genotyping have primarily focused on antidepressant medications (Groessl et al., 2018; Maciel et al., 2018), with no relevant studies on atomoxetine thus far.

### 9.3 Is there a cost effectiveness analysis of combined TDM and *CYP2D6* genotyping testing for atomoxetine?

For certain medications, integrating TDM with pharmacogenomics proves to be an effective approach to optimize treatment, emphasizing the importance of assessing the cost-effectiveness of both methods. One study found that a combined strategy of *NUDT15/TPMT* genotype screening prior to initiating azathioprine treatment, along with on-going TDM for management, was more cost-effective than alternatives that involved either genotyping *NUDT15* or *TPMT* alone or conducting genotyping without TDM in patients with inflammatory bowel disease (Zeng et al., 2021). However, comprehensive cost evaluations for atomoxetine are still lacking.

## 10 Perspectives and conclusions

Following the generic framework proposed by Beumer et al. (Beumer et al., 2019), we conducted a comprehensive literature review, evaluation, and summary to create a table (BOX 1) that prominently presents the critical questions of interest along with the evidence gathered to date. More importantly, we identified gaps in existing knowledge related to the goal of personalized dosing and identified areas for future research.

BOX 1 **| Summary of the critical questions and clinical evidence for the application of TDM.**

**Table:** 

Is there significant inter-individual variability in plasma concentrations using the current BW-based dosing regimen?
*Yes, inter-individual CL/F differences were high in the pediatric population, ranging from 14%–62% across reports*
Is there limited intra-individual variability in plasma concentrations?
*Only one study showed an intra-individual concentration variation of 21.3% for atomoxetine*
Do Drug-Drug Interactions (DDIs) impact the pharmacokinetic parameters of atomoxetine?
*Individuals taking atomoxetine along with a strong CYP2D6 inhibitor (e.g., bupropion, fluoxetine, and paroxetine) may experience higher than expected concentrations*
Is there a narrow therapeutic window?
*The recommended concentration reference range of 200–1,000 ng/mL is not a clear therapeutic window related to efficacy and tolerability*
Are there easy and clinically relevant biomarkers to predict response and/or toxicity at a given dose?
*CYP2D6 is the most potential biomarker to predict response and/or toxicity*
Is there an accepted and clinically relevant metric for systemic exposure to atomoxetine?
*Cmax is the recommended monitoring parameter to be associated with atomoxetine efficacy*
Is there evidence for the relationship between plasma atomoxetine concentration and clinical activity?
*Threshold Cmax above 268 ng/mL showed a good clinical efficacy of atomoxetine*
Is there evidence for the relationship between plasma atomoxetine concentration and tolerability?
*Limited studies have examined the relationship between plasma atomoxetine concentration and tolerability*
Is there evidence that TDM improves activity in patients receiving atomoxetine?
*No study compared the impact on the therapeutic effects of atomoxetine before and after the implementation of TDM.*
Is there evidence that TDM reduces tolerability in patients receiving atomoxetine?
*No study compared the impact on the tolerability of atomoxetine before and after the implementation of TDM.*
Are reliable assays available?
*Various bioanalytical assays have developed to analyze atomoxetine, like LC-UV, LC-MS, Nano-level assay*
Is the proper sampling timing and handling established?
*Consider measuring peak concentrations at 1–4 h time intervals based on CYP2D6 phenotype and activity score*
Is there a recommended therapeutic exposure range based on the clinical evidence?
*200–1,000 ng/mL is the recommended therapeutic range, with a good response of >268 or 400 ng/mL peak concentration*
Is there a dose-adaptation strategy?
*In cases of inadequate response and absence of side effects, CPIC guideline recommended to adjust the dose proportionally to approach 400 ng/mL*
Is there a cost effectiveness analysis of TDM testing for atomoxetine?
*No study has addressed this issue*
Is there a cost effectiveness analysis of CYP2D6 genotyping testing for atomoxetine?
*No study has addressed this issue*
Is there a cost effectiveness analysis of combined TDM and CYP2D6 genotyping testing for atomoxetine?
*No study has addressed this issue*

A fundamental aspect of achieving precision medicine is to distinguish a given patient from others with similar clinical presentations by combining genetic, biomarker, phenotypic, or psychosocial characteristics (Jameson and Longo, 2015). In this review article, we focus on personalized dosing of atomoxetine in children with ADHD, aiming to provide strategies for adjusting doses specifically for children who have been accurately diagnosed and are considered appropriate candidates for atomoxetine therapy. Our goal is to maximize therapeutic benefits while minimizing adverse reactions. In essence, we seek to determine the “right dose” for the “right person”. However, we still face numerous challenges.

### 10.1 Challenge 1: there is no established association between exposure and clinical response

The first challenge in implementing personalized dose adjustment lies in the unclear relationship between atomoxetine exposure levels and both its efficacy and adverse reactions. ADHD is a complex and heterogeneous disorder (Posner et al., 2020; LaBianca et al., 2024), highlighting the need to evaluate medication responses in relation to the pharmacokinetics and duration of action of the selected formulation. Optimal symptom management and functional improvement occur when blood levels of the medication are adequately maintained for the periods of greatest need and for the specific tasks at hand (Faraone et al., 2024). Current evidence generally supports identifying the lowest concentration or concentration range that ensures optimal efficacy; however, data regarding the maximum tolerable concentration levels still relatively sparse. Additionally, there is also scarce data on the relationship between exposure levels to atomoxetine and its adverse reactions. Consequently, there is no clear therapeutic window defined for atomoxetine. Without this defined window, there is no established “target value” for dose selection, complicating the process of making personalized dose adjustments. Also, the lack of a defined therapeutic window has compromised the role of PPK/PBPK models in predicting personalized doses, posing a significant challenge for future efforts in this area. In response to these challenges, machine learning-based predictive models have emerged as a promising strategy (Faraone et al., 2021; Faraone et al., 2022).

### 10.2 Challenge 2: there are no recognized predictors for atomoxetine therapy response

The second challenge involves the lack of predictors for treatment response to atomoxetine. By integrating biomarkers and clinical predictors of both response and adverse effects, clinicians could potentially tailor treatment for individual patients. However, there are currently no available clinical or biological predictors of response for ADHD (Buitelaar et al., 2022). At this time, the genotypes and phenotypes of CYP2D6 may serve as the most “reliable” predictor. As the primary metabolic enzyme for atomoxetine, CYP2D6’s metabolic activity directly influences the drug’s pharmacokinetic behavior, thereby linking exposure levels to both efficacy and adverse reactions. In other words, variations in CYP2D6 activity fundamentally “determine” the differences in both the efficacy and adverse reactions of atomoxetine. However, the predictive power of CYP2D6 is limited, mainly due to the lack of a well-established exposure-response relationship, as previously noted. Additionally, the inherent uncertainties in predicting drug response based solely on genetic markers, given the potential for false negatives or positives in *CYP2D6* genotyping, also pose challenges in clinical practice as well. Investigating ways to standardize the translation of genotyping data into actionable, evidence-based prescribing decisions is an important endeavor. Nonetheless, personalized dosing strategies that utilize CYP2D6 stratification remain crucial and significantly continue to the rational use of atomoxetine (Brown et al., 2019; Guo et al., 2024).

### 10.3 Challenge 3: quantitative assessment indicators for evaluating ADHD

The third challenge comes from how to objectively and accurately assess the clinical efficacy of atomoxetine (Wong et al., 2019; Raman et al., 2018). To tackle this issue, we require more objective and quantifiable indicators that can accurately depict changes in symptoms and reflect treatment outcomes. Currently, it is recognized that the diagnostic rate for female ADHD patients is lower than that for males (Martin, 2024). If this discrepancy continues to affect efficacy assessments, it could hinder the effective implementation of personalized dosing strategies.

### 10.4 Challenge 4: socio-political barriers to TDM and genotyping implementation

As early as 2015, it was clearly understood that achieving precision medicine would necessitate overcoming major challenges across various domains, including technological and socio-political aspects. TDM and pharmacogenomics, as key elements of precision medicine, face few technical hurdles; however, socio-political factors such as public support, affordability, and education pose even more obstacles (Kohane, 2015). Indeed, numerous challenges will persist in clinical practice, limiting the widespread implementation of precision medicine in clinical settings (Chenoweth et al., 2020).

While TDM and genotyping technologies do not inherently pose obstacles to implementing personalized medicine, their widespread use in clinical settings is not encouraging, particularly in children’s hospitals (Brown et al., 2021; Chenoweth et al., 2020; Just et al., 2019; Duarte et al., 2021), remains limited. Few institutions have the capability to conduct both TDM and genetic testing simultaneously, hindering individualized dose adjustments. For example, a nationwide survey by Jacob *et al.* found that only four centers implemented TDM for atomoxetine alongside CYP2D6 genotyping (Brown et al., 2021). Similarly, our recent survey on the clinical implementation of PGx testing revealed that only four children’s hospital conducted *CYP2D6* genotyping (Wu et al., 2024). Additionally, the cost associated with implementing genotyping and TDM in clinical practice raises significant concern for healthcare systems and patients, making cost evaluations critical (Morris et al., 2022). Furthermore, effective implementation also requires collaboration among genotyping and TDM labs, bioinformatics/IT for result analysis and communication, and clinicians for integrating patient care. Notably, only one center offered clinical decision support for atomoxetine and CYP2D6 interaction within its electronic prescribing system (Brown et al., 2021). Therefore, building multidisciplinary teams around personalized dosing is crucial (Barker et al., 2022); where such teams are in place, they can significantly enhance the effectiveness of personalized dosing strategies.

### 10.5 Challenge 5: comprehensive understanding of ADHD

Ultimately, a comprehensive understanding of the ADHD itself may present the greatest challenge. Our unwavering goal is to pursue personalized atomoxetine treatment, which depends on a deeper understanding of the condition’s heterogeneity through extensive studies into its etiology, pathophysiology, and clinical manifestations. Integrating multi-omics studies can facilitate the discovery and validation of biomarkers that could serve as potential clinical predictors of response (Hubers et al., 2024; Hagenbeek et al., 2023).

Additionally, it is essential to address medication adherence. Once adherence issues are resolved (Brikell et al., 2024), prioritizing the establishment of a precise match between patients and optimal atomoxetine treatment will become a focus for future research.

Overall, while this study extensively references various guidelines and theoretical frameworks, it lacks specific examples of clinical outcomes derived from these approaches. The key challenge is to effectively translate these theoretical concepts into practical, real-world clinical applications. Nonetheless, clinicians can gain valuable insights from the existing evidence, particularly in identifying the limitations of current guidelines and implementing personalized treatment across different clinical settings.
